# Increased uptake of doxorubicin by cells undergoing heat stress does not explain its synergistic cytotoxicity with hyperthermia

**DOI:** 10.1080/02656736.2019.1631494

**Published:** 2019

**Authors:** Anirudh Sharma, Sanem Özayral, Julia S. Caserto, Rosemarie ten Cate, Nicole M. Anders, James D. Barnett, Sri Kamal Kandala, Elizabeth Henderson, Jacqueline Stewart, Eleni Liapi, Michelle A. Rudek, Nicolaas A.P. Franken, Arlene L. Oei, Preethi Korangath, Fred Bunz, Robert Ivkov

**Affiliations:** a Department of Radiation Oncology and Molecular Radiation Sciences, Johns Hopkins University School of Medicine, Baltimore, MD, USA;; b Department of Radiation Oncology, Amsterdam University Medical Centers, University of Amsterdam, Amsterdam, Amsterdam, The Netherlands;; c Department of Oncology, Johns Hopkins University School of Medicine, Baltimore, MD, USA;; d Department of Mechanical Engineering, Johns Hopkins University, Baltimore, MD, USA;; e Department of Radiology and Radiological Sciences, Johns Hopkins Hospital, Baltimore, MD, USA;; f Institute for Nanobiotechnology, Johns Hopkins University, Baltimore, MD, USA;; g Department of Medicine, Johns Hopkins University, Baltimore, MD, USA;; h Division of Clinical Pharmacology, Johns Hopkins University, Baltimore, MD, USA;; i Department of Materials Science and Engineering, Johns Hopkins University, Baltimore, MD, USA

**Keywords:** Hyperthermia, chemotherapy, doxorubicin, colorectal cancer, thermal dose

## Abstract

**Purpose:**

A proposed mechanism for the enhanced effectiveness of hyperthermia and doxorubicin (Dox) combinations is increased intracellular Dox concentrations resulting from heat-induced cell stress. The purpose of this study was to determine whether specific varied Dox and heat combinations produce measurable effects greater than the additive combination, and whether these effects can be attributed to heat-induced increases in intracellular Dox concentrations.

**Methods:**

HCT116, HT29 and CT26 cells were exposed to Dox and water bath heating independently. A clonogenic survival assay was used to determine cell killing and intracellular Dox concentrations were measured in HCT116 cells with mass spectrometry. Cells were exposed to heating at 42 °C (60 min) and 0.5 μg/ml of Dox at varying intervals. Synergy was determined by curve-fitting and isobologram analysis.

**Results:**

All cell lines displayed synergistic effects of combined heating and Dox. A maximum synergistic effect was achieved with simultaneous cell exposure to Dox and heat. For exposures at 42 ° C, the synergistic effect was most pronounced at Dox concentrations <0.5 μg/ml. Increased intracellular concentrations of Dox in HCT116 cells caused by heat-stress did not generate a concomitant thermal enhancement.

**Conclusions:**

Simultaneous exposure of HCT116 cells to heating and Dox is more effective than sequential exposure. Heat-induced cell responses are accompanied by increased intracellular Dox concentrations; however, clonogenic survival data do not support this as the cause for synergistic cytotoxicity.

## Introduction

Colorectal cancer is the third leading cause of cancer-related death in the USA [1]. Doxorubicin (Dox) is one of many small-molecule chemotherapy agents used to treat cancers of the colon and other sites [2–4]. The biologic mechanism(s) responsible for the therapeutic benefits of Dox remain the focus of continuing study [5–18]; however, its use has been limited because of increased tolerance that manifests as either *de novo* or acquired chemoresistance [19–22].

Therapeutic hyperthermia (HT), defined as (clinical) heating to a range of 40–47 °C, can enhance treatment response and reduce recurrence when it is combined with other therapies [23–25]. These enhanced responses have been attributed to the biologic effects of heating or heat stress (HT), including inhibition of DNA damage repair via protein inactivation or denaturation [26,27]. Physiologic effects associated with increased temperature include blood perfusion and tumor oxygenation, which are also thought to contribute [28]. Regardless of mechanism or mode of action, improved outcomes and reversed chemoresistance often accompany treatment combinations that include HT [29–39]. Cytoreductive surgery has been effectively combined with post-operative hyperthermic intraperitoneal chemotherapy (HIPEC) for various peritoneal neoplasms including metastatic colorectal cancer [40,41]. HIPEC offers an advantage by combining favorable pharmacokinetics of intra-cavitary delivery of a pre-heated chemotherapy solution with the enhanced cytotoxicity of heat to achieve a prolonged disease-free and overall survival [42].

HIPEC-based therapy is administered by circulating a heated chemotherapeutic solution through the peritoneal cavity of the patient in a peri-operative setting [42]. For certain drug combinations, the optimal schedule may be one other than simultaneous administration. Studies that compare the effectiveness of various therapy combinations using cell culture models provide clues to optimize HIPEC and other combination therapies [32–36].

A systematic examination of potency related to treatment schedule for Dox + HT combinations is lacking. There is also a continuing debate whether the enhancement by HT can be attributed to heat-induced changes of cellular pharmacodynamics such as enhanced intracellular uptake of Dox (i.e. heat induced membrane permeabilization or altered cell metabolism) versus enhanced toxicity from heat-induced DNA-repair inhibition, or both [32,33,43–45].

The objective of this study was to determine the optimal schedule for Dox + HT combinations in vitro and to determine whether the potency results from a heat-related increased intracellular concentration of Dox. Here, we demonstrate that a simultaneous combination of HT with Dox produces the most potent synergistic cytotoxicity in colorectal cancer cells—HCT116, HT29 and CT26. We also determined in HCT116 cells that the heat-related increase of intracellular Dox does not alone account for the observed synergistic interaction.

## Materials and methods

### Cells and reagents

#### HCT116

Human colorectal cancer cells HCT116 (HTB-81, American Type Culture Collection (ATCC), Rockville, MD) and HT29 (HTB-38, ATCC), were grown and maintained in T25 flasks (Corning, Inc. Corning, NY) with McCoy’s 5 A medium supplemented with 10% fetal bovine serum (FBS) (Gibco Laboratories, Lenexa, KS) and 1% Pen/Strep (Penicillin/Streptomycin) in a humidified incubator at 37 °C and 5% CO_2_. Cell line authentication was conducted by the Johns Hopkins School of Medicine Genetic Resources Core Facility (GRCF) using short tandem repeat analysis and matched against ATCC and Deutsche Sammlung von Mikroorganismen und Zellkulturen databases to ensure the genetic origins. Mycoplasma testing was conducted by the GRCF using a PCR-based commercial detection kit. Data and reports provided upon request.

#### HT29

Human colorectal cancer cells HT29 (HTB-38, ATCC), were grown and maintained similarly to HCT116, except no penicillin/streptomycin was used.

#### CT26

Murine colorectal cancer cells CT26 (ATCC CRL-2639) were grown in RPMI with 10% FBS (heat inactivated), 2 mM L-glutamine, 1 mM sodium pyruvate, non-essential amino acid, 0.05 mM 2-mercaptoethanol and 1% Pen/Strep in a humidified incubator at 37 °C and 5% CO_2_.

Doxorubicin-HCl (Dox) (Pfizer, New York, New York) was received as an injectable formulation with concentration 10 mg Dox/5 ml saline solution. It was diluted to 0.5 mg/ml with PBS and aliquots were stored at 4 °C.

### Water bath calibration and thermometry

For CT26 and HCT116 cells, a circulating water bath (Polyscience AD15H200, Niles, IL) was used for all studies. Water bath temperatures were monitored with a FISO optical probe (FISO Inc., Quebec, Canada), calibrated against a National Institute of Standards and Technology-traceable mercury thermometer (Thermco Products Inc, Lafayette, NJ). To monitor the temperature during HT experiments, a calibrated FISO optical probe was placed into a surrogate T25 flask containing 5 ml media (no cells). For each experiment, the surrogate flask was lowered into the water bath simultaneously with experimental flasks. The offset between the temperature inside the flask and outside the flask (water bath) was measured and used to determine water bath temperature set point so that the internal flask temperature was within ±0.3 °C of the target temperature, with an average warm-up time of ~20 min and cool-down for ~2 min. Temperatures were recorded during warm-up, exposure at target temperature, and cool-down to 37 °C. Reported temperature variances represent recorded experimental variation. Measurement variance was significantly lower (< ±0.05 °C).

For HT29 cells, HT was performed by partially submerging T25 flasks in a thermostatically controlled circulating water bath (Lauda auqualine AL12, Beun de Ronde, The Netherlands). Temperature was monitored in a surrogate T25 flask containing medium only with thermoprobes (Ellab, Denmark). Cells were heated in a 5% CO_2_/95% air atmosphere and air inflow of 2 l/min. All temperatures were recorded during an ~2 min warm-up time to reach the desired temperature (±0.1 °C), a 60 min-treatment and a maximal 5 min cool-down to 37 °C.

### Dox, heating and combination experiments

**Figure 1.**
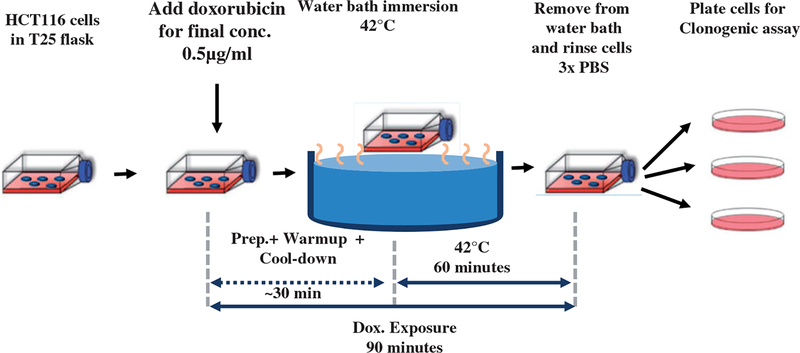
Schematic diagram of experiment design to evaluate effectiveness of combined heat-stress (HT) and exposure to Dox of human colorectal cancer cells. See text for details.

A schematic of the experiments is provided in Figure 1. For all experiments, 250 000 cells were plated in 5 ml media in a T25 flask and placed into the incubator at 37 °C for 48 h. A clonogenic assay was used to determine surviving fraction as the end point to assess effectiveness. Each experiment had three or more replicates and every experiment was repeated at least three times.

Single-agent dose escalation studies were performed with Dox and HT individually. In addition, three combination exposure experiments were performed: (1) Dox followed immediately by HT (sequential, HCT116); (2) HT followed immediately by Dox (sequential, HCT116); and (3) simultaneous Dox + HT (all cell lines). Following all exposures, cells were washed three times with 5 ml PBS and harvested by incubating in 1 ml 0.05% trypsin for 3 min. Trypsin was neutralized with 3 ml media and cells were counted using a cell counter (Cellometer Auto T4, Nexelcelom Bioscience) and plated for clonogenic assay.

Depending on experiment, single-agent Dox concentrations were 0, 0.1, 0.25, 0.5, 1 or 1.25 μg/ml and cells were incubated at 37 °C for 90 min. Dox-containing media was aspirated and cells were washed three times with PBS to remove extracellular Dox. Cells were collected and centrifuged at 1200 rpm for 5 min to form pellets. Cell pellets were then re-dispersed in fresh media, counted and plated.

For single-agent HT experiments, cells were exposed to increasing thermal dose by adjusting and maintaining water bath temperatures at 37, 41, 42, 43, 44 and 45 °C, respectively for 60 min. Flasks were covered with parafilm to maintain sterility inside the flask, then immersed into the water bath and treated for 1 h at temperature. Total time including sample processing, warm-up and cool-down was 90 min, thus time of exposure was held constant and temperature was varied. Cells were then rinsed three times with PBS, counted and plated for clonogenic assay as above.

**Figure 2.**
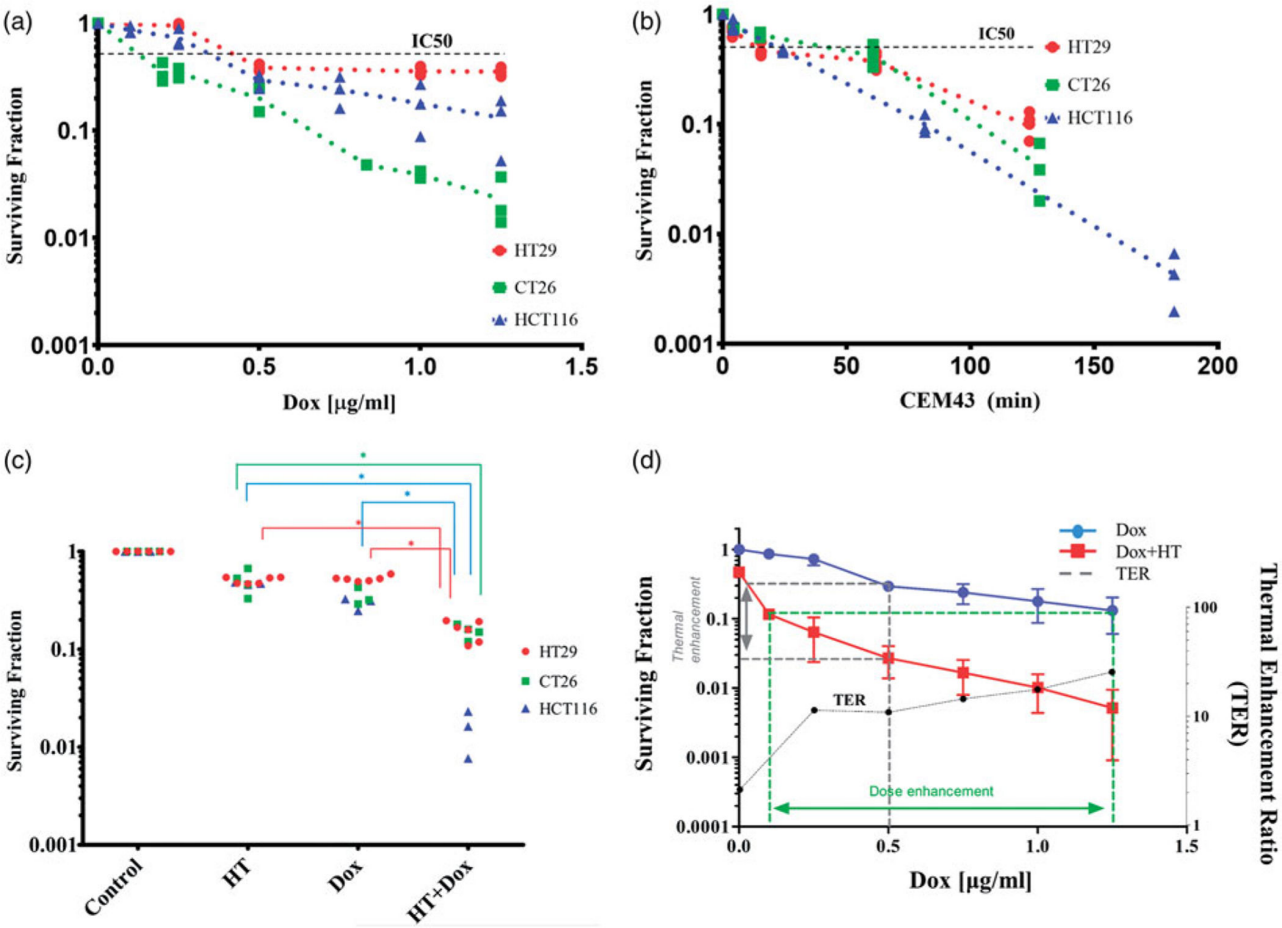
(a) Clonogenic survival of HCT116 (▲), HT29 (●) and CT26 (■) cells following single agent HT with escalating thermal dose (CEM43) normalized to 37 °C controls (red) and (b) clonogenic survival of HCT116 (▲), HT29 (●) and CT26 (■) cells following Dox exposure for 90 min at 37 °C normalized to negative controls. Scatter points values obtained from at least three separate experiments. For each experiment, there were three technical replicates for evaluation of clonogenic survival. (c) Univariate scatterplot showing measured clonogenic survival of HCT116 (▲), HT29 (●) and CT26 (■) cells following varied exposures to HT and Dox combinations. For each, individual data points are plotted following exposure to 60 min HT and dox for 90 min. From various sequences of heat-stress and Dox administration, simultaneous application of both modalities yielded the greatest cytotoxicity. (*p < .003, one-way ANOVA with post-hoc Tukey’s multiple comparisons test, Supplementary Figure S4). (d) Measured clonogenic survival of HCT116 cells following 90-min exposure to Dox at the indicated concentrations and at 37 °C (blue) repeated from (a). The red curve indicates clonogenic survival of HCT116 cells in a combination exposure where each data point represents a mean of several replicate experiments with fixed Dox concentration and temperature of 42 °C for a total drug-exposure time of 90 min and heat exposure time of 60 min. The green dotted lines indicate the DEF of combination exposure over Dox at 37 °C to achieve the same cytotoxicity. At IC90, the DEF is ~12.5. The gray dotted line indicates the TER for a fixed dose of Dox. At 0.5 mg/ml, Dox the TER is ~11. TER vs Dox concentration (μg/ml) (black dotted line) shows that TER increases gradually for Dox concentrations ≥ 0.25 μg/ml, y-axis is on the right.

For all Dox + HT combination experiments, Dox concentrations and HT thermal doses were chosen as approximately, the IC50 condition (Figure 2(a) and Table S1). For Dox + HT (simultaneous) experiments in HCT116, for example, 5 ml of pre-warmed (37 °C) media containing Dox at 1 lμ/ml concentration was added to the 5 ml of existing media in the T25 flask, so that the final concentration of Dox in the flask was0.5 lg/ml. Immediately following Dox addition, the flasks were immersed into the water bath with the surrogate flask containing 10 ml of media and treated for 60 min at steady-state temperature of 42 °C ± 0.3 °C. This process of adding the Dox to the flask and immersing in the water bath was ~1 min. The 90-min exposure to Dox began when Dox was added to the flask. Total exposure time to Dox + HT, including, warm-up, cool-down and sample processing was 90 min. Following exposure, cells were prepared for clonogenic assay as described below.

### Clonogenic survival assay

Serial, 10-fold dilutions were prepared from treatment and control flasks and cells were re-counted with each dilution. Fixed numbers of cells were plated in 10 mm dishes. Cells plated for clonogenic assay following each treatment are listed in Table S2. Cells were incubated for 10 days (7 days for CT26) and stained with 2% crystal violet. Colonies having more than 50 cells were counted. Mean plating efficiency was determined from untreated control samples to be 63% (range 60–70%). Surviving fraction was determined relative to controls and one-way ANOVA with post-hoc Tukey’s multiple comparisons test was conducted to compare statistical significance [46].

### Thermal dose calculations

Thermal isoeffect dose, defined as cumulative equivalent minutes referenced to 43 °C (CEM43) was used to provide a comparison of time-at-temperature combinations among the various treatment conditions. It is calculated using the following formula:

(1) $$\text{CEM}43\;(\text{min})=\sum_{i=1}^{n}t_{i}*R^{\left(43-T_{i}\right)}$$

where CEM43 is a normalization of experimental time-at-temperature to equivalent time at the reference temperature 43 °C, *T*_*i*_ is average temperature at time interval *t*_*i*_*, R* = 0.428 (*T* > 43 °C) and *R* = 0.233 (*T* < 43 °C) for human cells [47]. Temperature-time data recorded from the surrogate flasks were used to calculate thermal isoeffect dose (Figure S1). Temperature and time-at-temperature data obtained for all cell lines, along with calculated CEM43 values are provided in Table S3. Slight variations among CEM43 values among the experiments arising from differences in warmup or cool-down times are noted.

### Mass spectrometry analysis of intracellular dox

Following exposure, HCT116 cells were washed three times with PBS and collected using methods described above. From each experiment 500 000 cells were collected in triplicate, spun at 5,000 rpm for 10 min in a 4 °C centrifuge. Subsequently, cell pellets were flash dried using liquid nitrogen. Cells were lysed and analytes were extracted with methanol. Concentrations of recovered analytes, Dox and doxorubicinol, were measured with a liquid chromatography tandem mass spectrometer (5500, SCIEX, Framingham, MA) over the calibration range of 5–1000 ng/ml.

### Analysis of dox and thermal dose data

Weighted least-squares fitting was performed using OriginPro (OriginLab Corporation, Northampton, MA) and GraphPad Prism (GraphPad Software, LaJolla, CA) to fit a function to clonogenic survival data (Figure S2). For HCT116 cells, the Exp2PMod1 model was used to fit to both data sets and is described by the formula [48]

(2) S=a*exp (bx)

Where, *S* is the surviving cell fraction, *x* is concentration or dose of cytotoxic agent, and *a* and *b* are fitting parameters. For Dox, fitting was performed for the concentration range 0.5–1.25 μg/ml to determine IC 90 value (90% cell kill or 10% clonogenic survival). For heat-shock, the entire thermal dose range was used to determine IC 90 (HCT116) value. Similarly, for HT29 and CT26 data sets were fitted and IC 85 doses were extracted. Equations used are summarized in Table S3 and curve-fits are shown in Figure S4.

### Measures of effectiveness of combined exposure: DEF, TER and isobologram analysis

A dose enhancement factor (DEF) is defined for the same surviving fraction as:

(3) $$\text{DEF}=\frac{\text{ Dox dose without HT }}{\text{ Dox dose with HT}}$$

Where, DEF represents the factor by which effectiveness of one agent changes relative to its combination with another. In the present case, the DEF represents the concentration of Dox required to achieve the same effectiveness of a specific combination of Dox + HT. Stated another way, it is a measure of the reduced chemotherapy concentration of the combination to achieve the same biological response as single agent [48].

The thermal enhancement ratio (TER) is a measure of the chemosensitivity at 37 °C relative to the sensitivity at elevated temperatures [49]. TER is defined for the same Dox concentration as:

(4) $$\text{TER}=\frac{\text{ Clonogenic survival without HT }}{\text{ Clonogenic survival with HT}}$$

**Figure 3.**
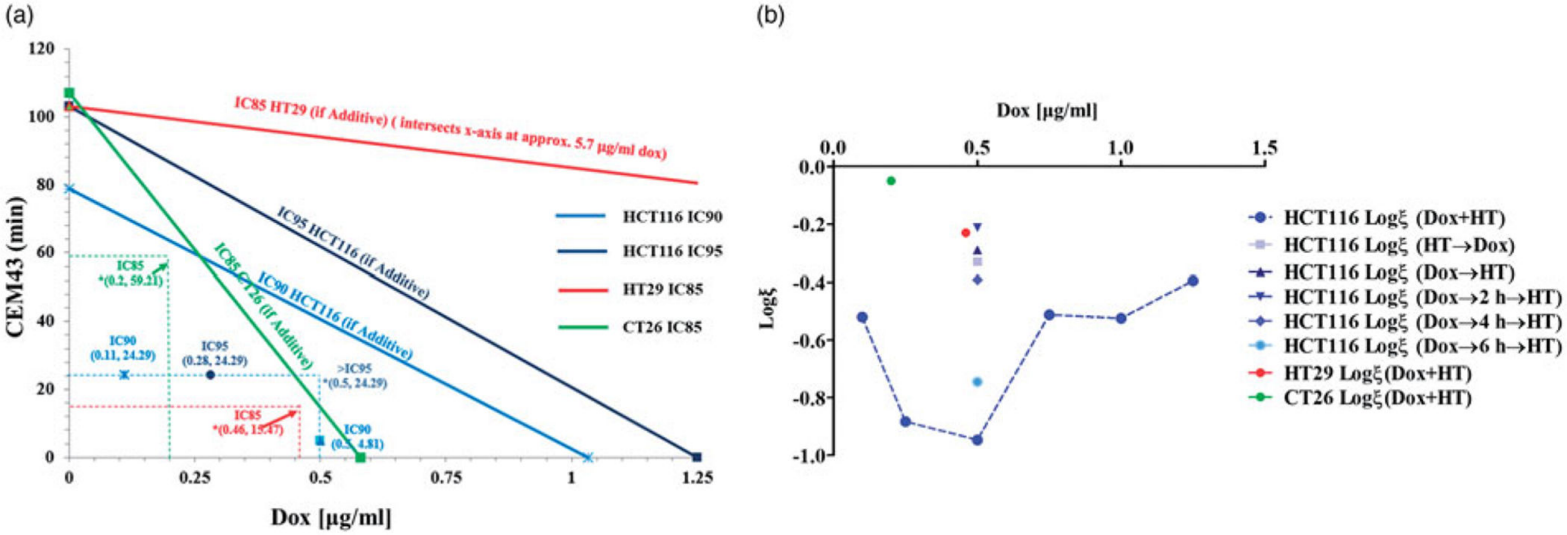
(a) Isobologram analysis of Dox dose escalation at 42 °C (●) and thermal dose escalation at 0.5 μg/ml Dox (▲) to determine whether combined exposure was synergistic, additive, or antagonistic. The lower left region (origin) of the IC90 line is the synergistic region and the upper right region represents antagonistic interactions. All data points that lie on the line indicate additive effects of the combination. Combined exposure to Dox and heat stress produces effects, measured by clonogenic survival, that indicate synergistic interactions. *(x,y) represent Dox dose and HT CEM43 dose used in combination treatments. Since all combinations tested lie on the origin side of the isobolograms, this indicates that these combination treatments are synergistic. (b) Interaction coefficient, *ξ*, vs Dox concentration for combination experiments. *ξ* provides a method for quantitatively comparing the level of synergy in various combination treatments. The plot shows highest synergy for simultaneous application of HT and Dox. Dox concentration was 0.5 μg/ml. Corresponding univariate scatter plot showing mean surviving fraction for various combination treatments are provided in Supplementary Figure 3.

Isobolograms are graphical iso-effect representations used to assess the interaction between two drug or treatment modalities [48]. A graph is constructed on a coordinate system on which individual drug concentrations form the axes. A ‘line of additivity’ divides the x-y plane into additive, synergistic and antagonistic regions of interaction allowing one to distinguish the interaction between two agents by inspection [48]. MS Excel (Microsoft Corp.) was used to tabulate and plot values of IC90 to determine the relationship between Dox and HT on the response of HCT116 cells. IC90 values from thermal dose escalation and Dox dose escalation were used as intercepts on the *y*-axis and *x*-axis, respectively to define the IC 90 line in the isobologram. The combination exposure is classified as synergistic if the IC 90 value lies on the origin side of the IC90 line. Similarly, IC 85 dose values were compared to the IC 85 additive lines for HT29 and CT26 cell lines to determine synergy. Figures S6 displays clonogenic survival data plotted with Δ*T* and *e*(Δ*T*) (inset) on the abscissa, and Figure S7 displays isobologram analysis with Δ*T*. These representations are equivalent to those presented in Figures 2(b) and 3(a), respectively because the time of exposure was fixed at 60 min in all cases.

## Results and discussion

All cancer cells studied here exhibited a dose-dependent toxicity to Dox exposure, as measured by clonogenic survival, within the Dox concentration range 0–1.25 μg/ml. The IC50 values for all cells were ≤0.5 μg/ml. CT26 cells were the most sensitive and HT29 cells were the least sensitive to increasing concentrations of Dox exposure, respectively. The measured surviving fraction of HCT116 cells appeared to decrease exponentially with exposure to single-agent Dox at concentrations >0.5 μg/ml (blue symbols), Figure 2(a).

In HT only thermal dose escalation (i.e. 60-min exposure with increasing temperature) treatments, CT26 cells appeared to be least sensitive to heat exposure within the measured range, while the surviving fraction of HCT116 cells appeared to decrease exponentially with increasing thermal dose (Figure 2(b)). Results of curve fitting are provided in Figures S2 and S3 and Table S4.

The isoeffect dose metric, CEM43 has its roots in measurements of cellular response to heat stress by clonogenic survival assays in cultured cells [46,47,50–64]. The physical and biological underpinnings governing CEM43 calculations are based upon an empirical Arrhenius relationship of surviving fractions for cell populations that are observed to hold for exposures at temperatures ~42 °C–47 °C, and for relatively short duration (<120 min). Clonogenic survival assays measure reproductive cell death, i.e. replication failure, and thus they do not directly measure cytotoxicity [46]. In the decades since CEM43 was first proposed, it has been used and misused to compare biological response to HT for varied time-temperature combinations, and across multiple assay platforms [64]. Its relevance to translational studies in animal models and clinical applications continue to be debated, as does its broader applicability across cell lines among species and even for cells derived from tissues within a single species [50–64]. Given its origins in tissue culture experiments, using clonogenic survival as the endpoint assay, its applications and use ought to be restricted to these experimental conditions, and interpretation of results should be undertaken with care. Furthermore, for extreme temperatures (>~47 °C) and long exposure times (~>90–120 min), the validity of CEM43 becomes a question because other biological responses, e.g., cell lysis, ablation, thermal tolerance, etc.) at higher thermal doses become relevant and even dominate, thus invalidating the assumptions and rationale forming the foundation of the CEM43 metric [51,56,64].

For studies reported here, we restricted exposure times of cells in culture to 60 min, thus comparisons of thermal dose are measured by clonogenic assay to varying temperatures within the accepted HT limits 41 °C–45 °C (Table S3). For comparisons of effect of combined Dox + HT combinations, temperature-time exposure was fixed at 42 °C and 60 min. Thus, within these strict limitations, use of CEM43 is reasonable as a metric for comparing within an individual cell line. Nevertheless, given questions regarding clinical applicability of CEM43, we provide temperature-referenced data for Figures 2(b) and 3(a) in Figures S6 and S7. We note that conclusions are unchanged regardless of CEM43- or temperature-referenced data in the context of experimental conditions reported here.

Exposure to combined Dox + HT, regardless of cell-line, was more effective than either single agent; however, HCT116 cells appeared to show the greatest sensitivity to Dox + HT treatments (lowest surviving fraction) relative to single-agent treatments. Thus, HCT116 cells appeared to be the most interesting cell line to study comparisons of sequential Dox + HT combinations, and intracellular concentrations of heat-stress induced Dox uptake. Dox + HT sequences were varied with HCT116 cells to ascertain the effect of treatment schedule on surviving fraction. No difference between the two sequential exposures was observed (Figure S4). On the other hand, simultaneous exposure to combined Dox + HT proved more effective than either sequential combination tested (Figure S4).

Simultaneous exposure of cells to HT (42 °C) with varying Dox concentrations was compared against single agent Dox for HCT116 cells (Figure 2(d)). Most notable was the dramatic decrease of surviving fraction with Dox concentration in the range 0–0.25 μg/ml; however, for Dox concentrations >0.25 μg/ml further increases were modest suggesting an optimal exposure of ~0.25 μg/ml. This relationship was quantified by TER values calculated from surviving fraction data using Equation (5), and results are displayed in Figure 2(d). As anticipated by raw surviving fraction data, the simultaneous combination of Dox + HT produced the most significant rise of TER with media Dox concentrations up to 0.25 μg/ml. Further Dox addition to media produced only modest incremental increases of TER.

Results of isobologram calculations for all cell lines studied are shown in Figure 3(a) and S7. For the IC90 condition and for a media Dox concentration of 0.5 μg/ml, the effects of combined Dox exposure with heat at 42 °C showed the interaction is synergistic. For all other combination exposures, we obtained the same synergistic outcome (Figures 3(b) and S7), but with varying magnitude. The interaction can be quantified by defining an interaction coefficient,

(5) $$\xi =\frac{\left(\text{ SF Combination }\right)}{\left(\text{ SF treatment }1)*(\text{ SF treatment }2\right)}$$

where SF = surviving fraction measured from clonogenic assay was used. Thus, log(*ξ*) for a synergistic combination yields a negative value with increasing magnitude for more synergistic interactions.

**Figure 4.**
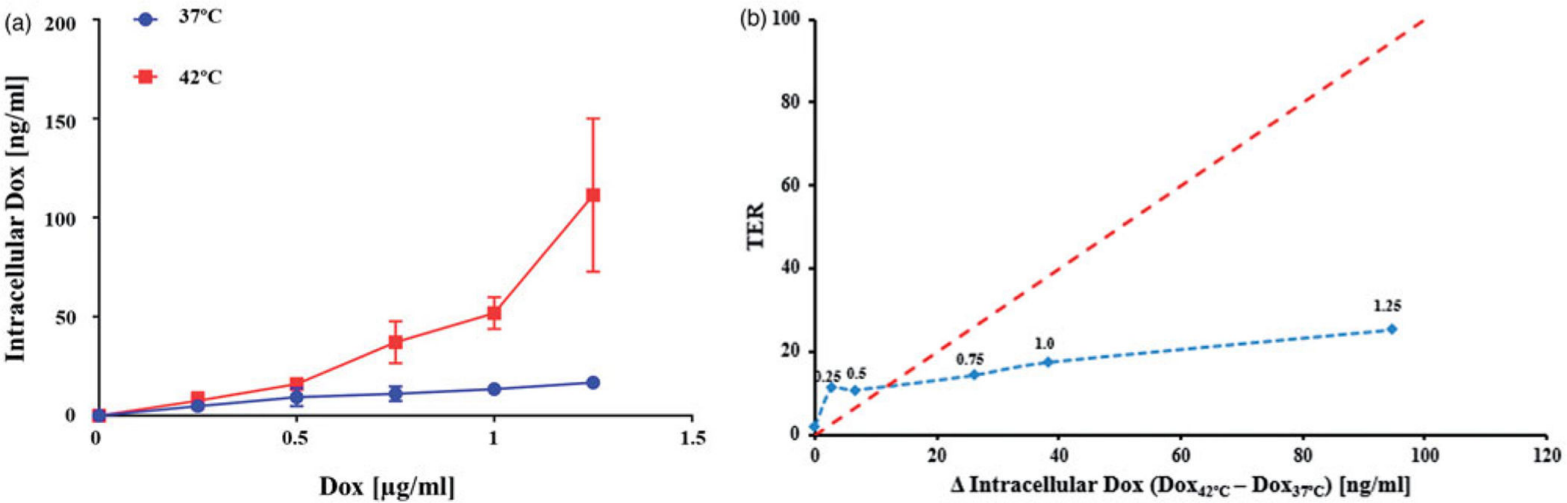
(a) Concentration of Dox (ng/ml) recovered from HCT116 cells using mass spectrometry for exposure to various media concentrations of Dox at 42 °C. Exposure to Dox in media having concentrations 0.75, 1 and 1.25 μg/ml. Exposure to Dox at 42 °C yielded higher recovered Dox than corresponding exposures at 37 °C (*p* ≤.04 Welch’s *t*-test, *N* = 3 independent experiments). (b) TER vs change of recovered (intracellular) Dox at 42 °C relative to 37 °C shows increased in intracellular Dox did not generate a comparable increase in TER. The red dotted line is a reference drawn to indicate a hypothetical 1:1 behavior. The blue dotted line is a visual aid. Numbers above data points indicated the Dox concentration in media (μg/ml).

The amounts of Dox recovered from (HCT116) cells exposed to Dox in media were relatively similar at either temperature for media Dox concentrations up to 0.5 μg/mL (Figure 4(a)). However, significantly more intracellular Dox was recovered from cells exposed to Dox concentration >0.5 μg/mL when they were simultaneously heated at 42 °C than was recovered from cells exposed to the same Dox concentration at 37 °C (*p* ≤ .04, with Welch’s *t*-test, *N* = 3 independent experiments). The metabolite doxorubicinol was undetectable in all conditions. Plotting TER against the difference in intracellular Dox shows that a large increase in Dox uptake was accompanied by only a modest increase in TER (Figure 4(b)). This result demonstrates that increased intracellular Dox accompanying cell heating is an unlikely explanation for the synergistic benefit of the heat + Dox combinations in HCT116 cells, which exhibited the greatest synergistic effect of Dox + heat.

One of the proposed mechanisms for Dox-induced cytotoxicity involves generation of semi-quinone free radicals from the quinone structure of Dox, which may induce free-radical damage to DNA or may facilitate formation of super-oxides, hydroxyl radicals and peroxides, which in turn, damage the DNA [9,10,43]. Our findings show that increased intracellular uptake of Dox alone does not explain the observed synergistic effects of combination treatment (Dox + HT), suggesting that an alternate mechanism of synergistic toxicity is involved. Indeed, several studies show that HT can increase intracellular ROS generation, when used in combination with ROS-modulating drugs [65–67]. As Dox is known to increase ROS generation in cells [9,43], heat used in combination can increase ROS levels by similar mechanisms and through acceleration of reaction rates that involve generation of free radicals from Dox. Future work would empirically test this hypothesis by measuring ROS levels through standard assays [65]. Differences in responses to combination treatments between normal and cancer cells can be expected, as previous literature indicates [68,69], allowing differential treatment of tumor vs non-tumor tissues.

Additionally, our findings indicate that both sequential (HT→ Dox) and (Dox→HT) treatments, while synergistic when administered within a short time, had similar potencies (Figure S4(a)), suggesting the mechanism may be independent of the sequence. The effect of schedule during sequential combination treatments should be further investigated by modulating the time gap between the administration of HT and Dox (Supplementary Figure S4(b)). Preliminary results suggest that a detailed mechanistic study should take into consideration sensitivity of the treatment to scheduling intervals (Figure S4). Such an oxidative metabolic mechanism presents an interesting avenue for exploration of combined effects of HT with Dox and other chemotherapy drugs for HIPEC and other applications [66].

Further study is warranted with additional cell lines; however, based on these results, we suggest that the biological mechanism(s) driving the observed synergy between HT and Dox may require a relatively low threshold level of intracellular Dox beyond which additional increases in concentration provide little gain in cell killing. A deeper understanding of these relationships could help guide optimization of clinical regimens to maximize efficacy and minimize toxicity.

## Summary and conclusions

It has been recognized for several decades that HT improves the effectiveness of many cancer chemotherapy agents [28,32–37]. Here, we demonstrated with three cell lines that combined Dox + HT results in synergistic cytotoxicity. The increased cytotoxicity of Dox when administered with HT has been attributed, in part, to an increased heat-induced membrane permeability and concomitant cellular uptake of Dox [32,33,44]. We demonstrate here with HCT116 human colorectal cancer cells, a line displaying the greatest synergistic effect, that an increased cellular uptake of Dox indeed accompanies transient HT. However, this effect does not explain the measured synergy of the combined exposure because increased toxicity is only modest or incremental at the higher measured intracellular Dox concentrations. Stated another way, increased intracellular Dox, beyond a certain threshold, may not generate a proportional therapeutic enhancement. We speculate that another biological mechanism, perhaps related to DNA-damage repair, dominates the measured synergistic benefit of HT and Dox combinations. Finally, our combination Dox + HT experiments demonstrated the superiority of simultaneous exposure to these two agents. These may be important and potentially related clues to help identify the mechanistic effects of HT and Dox combinations, which can be used to benefit clinical optimization.

## Supplementary Material

Suppl 1
