# Supplementary material for: Increased uptake of doxorubicin by cells undergoing heat stress does not explain its synergistic cytotoxicity with hyperthermia
Source: Int J Hyperthermia. Author manuscript; Available in PMC 2019 Dec 27. (PMC6934043; doi:10.1080/02656736.2019.1631494)
Supplement: Suppl 1 [file NIHMS1063462-supplement-Suppl_1.pdf]

Supplementary Materials
Heat-shock induced intracellular doxorubicin concentration increase does not explain synergistic cytotoxicity

Anirudh Sharma, Sanem Özayral, Julia S. Caserto, Rosemarie ten Cate, Nicole M. Anders, James D. Barnett, Sri Kamal Kandala, Elizabeth Henderson, Jacqueline Stewart, Eleni Liapi, Michelle A. Rudek, Nicolaas A.P. Franken, Arlene L. Oei, Preethi Korangath, Fred Bunz, and Robert Ivkov

Table S1: Dox and HT doses in combination treatments for all cell lines.

**Cell line**	**HT Steady-state Temperature (^{0}C)**	**CEM43 (mins) Mean ±SD**	**Dox dose (µg/ml)**
HCT116	42	24.3±3.2	0.5
HT29	42	15.5±0.1	0.46
CT26	43	57. 7±4.7	0.2

Notes: Dox refers to a doxorubicin dose; HT refers to heat-shock treatment at listed temperature; Dox+HT refers to simultaneous doxorubicin treatment and heat-shock.

Table S2: Plated cell numbers in 10 mm dish following each treatment in T25 flask for clonogenic survival.

Untreated	Doxo.	HT	Dox+HT	Dox→HT	HT→Dox
300	1000	1000	5000	1000	1000
1000	2000	2000	10000	5000	5000
	5000	5000	30000	10000	10000

Table S3: Mean thermal dose CEM43 value vs temperature and exposure time for each cell line.

**CEM43 (mins)**	**Temperature (°C)**	**Time (mins)**
	**HCT116**	
4.12	41	60
24.29	42	60
81.63	43	60
182.3	44	60
	**HT29**	
3.85	41	60
15.44	42	60
61.94	43	60
123.85	44	60
	**CT26**	
4.4	41	60
15.1	42	60
60.67	43	60
127.94	44	60

Table S4: Fitting equations used in weighted-least squares fit of HT and Dox dose escalation data for each cell line.

Cell line	Fitted equation<br>Dox	Fitted equation<br>HT
HCT116	$S = a * e^{bx}$<br>S, survival fraction<br>a,b, fitting constants<br>x, dose	$S = a * e^{bx}$<br>S, survival fraction<br>a,b, fitting constants<br>x, dose
HT29	$S = b + \frac{\{(a-b) * Frac\}}{[1 + 10^{\{(LogEC50_1 - x) * nH1\}}]} + \frac{\{(a-b) * (1 - Frac)\}}{[1 + 10^{\{(LogEC50_2 - x) * nH2\}}]}$<br>"Biphasic" response<br>S, survival fraction<br>a, maximal S (top plateau)<br>b, minimum S (bottom plateau)<br>nH1 and nH2 are unitless slope factors<br>Frac is proportion of maximal response due to the more potent phase (Refer to Graphpad Prism).<br>LogEC50_1 and LogEC50_2 are the concentrations that give half-maximal stimulatory and inhibitory effects, measured in the same units as x (the dose).	$S = e^{a+bx+cx2}$<br>S, survival fraction<br>a,b,c fitting constants<br>x, dose
CT26	$S = b + \frac{a-b}{[1+10^{\{(LogIC50-x)*m\}}]}$<br>S, survival fraction<br>a, maximal S (top plateau)<br>b, minimum S (bottom plateau)<br>m, hill slope<br>x, dose	$S = e^{a+bx+cx2}$<br>S, survival fraction<br>a,b,c fitting constants<br>x, dose

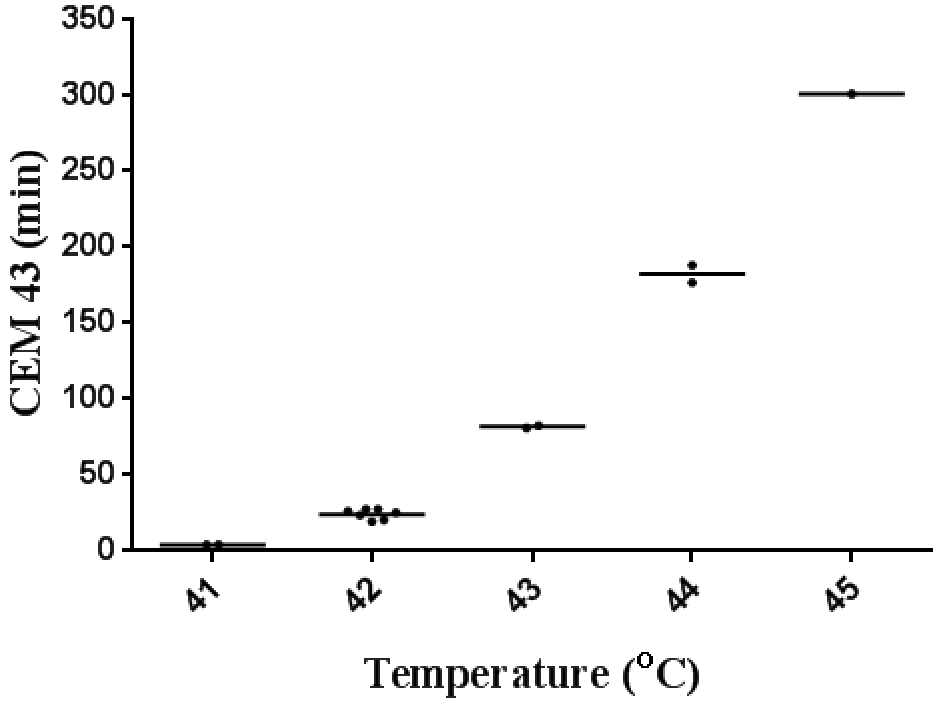

Figure S1: Thermal dose vs temperature scatter plot for HCT116 cells.

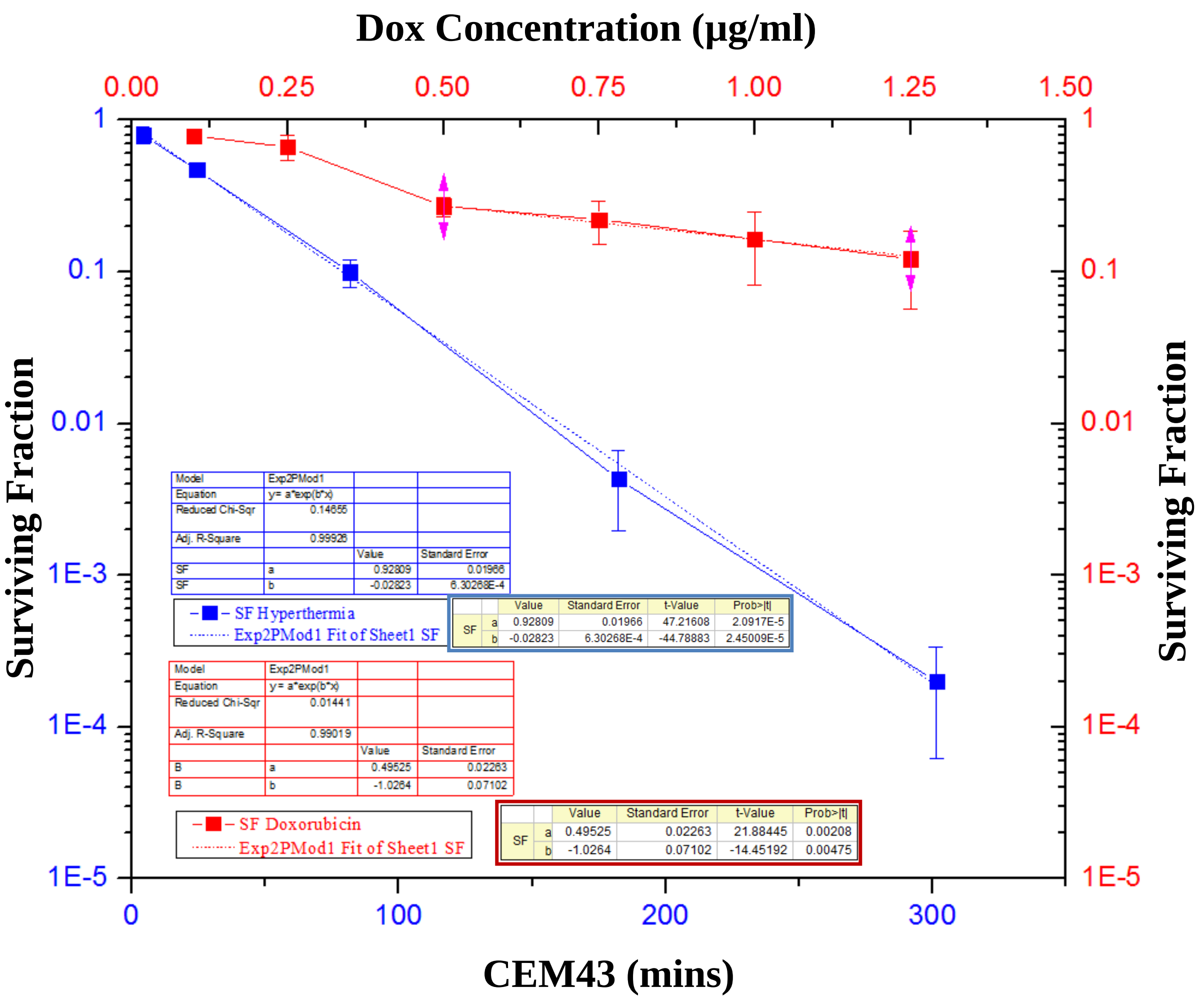

Figure S2: Weighted least squares fitting of clonogenic survival data with Dox only dose escalation (red) and HT only dose escalation (blue) for HCT116 cells.

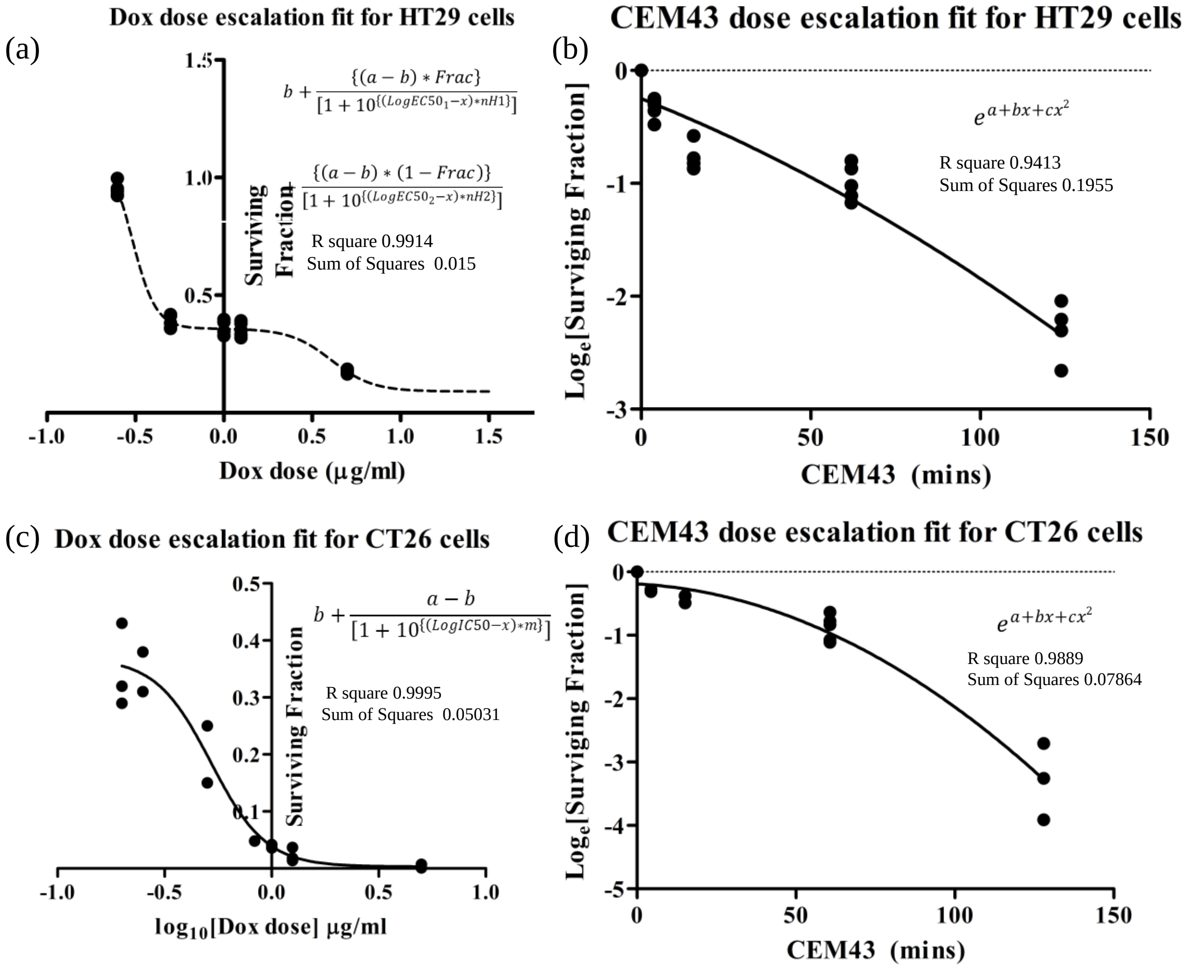

Figure S3: Curve fitting of Dox only and HT only treated HT29 (a and b) cells and CT26 cells (c and d).

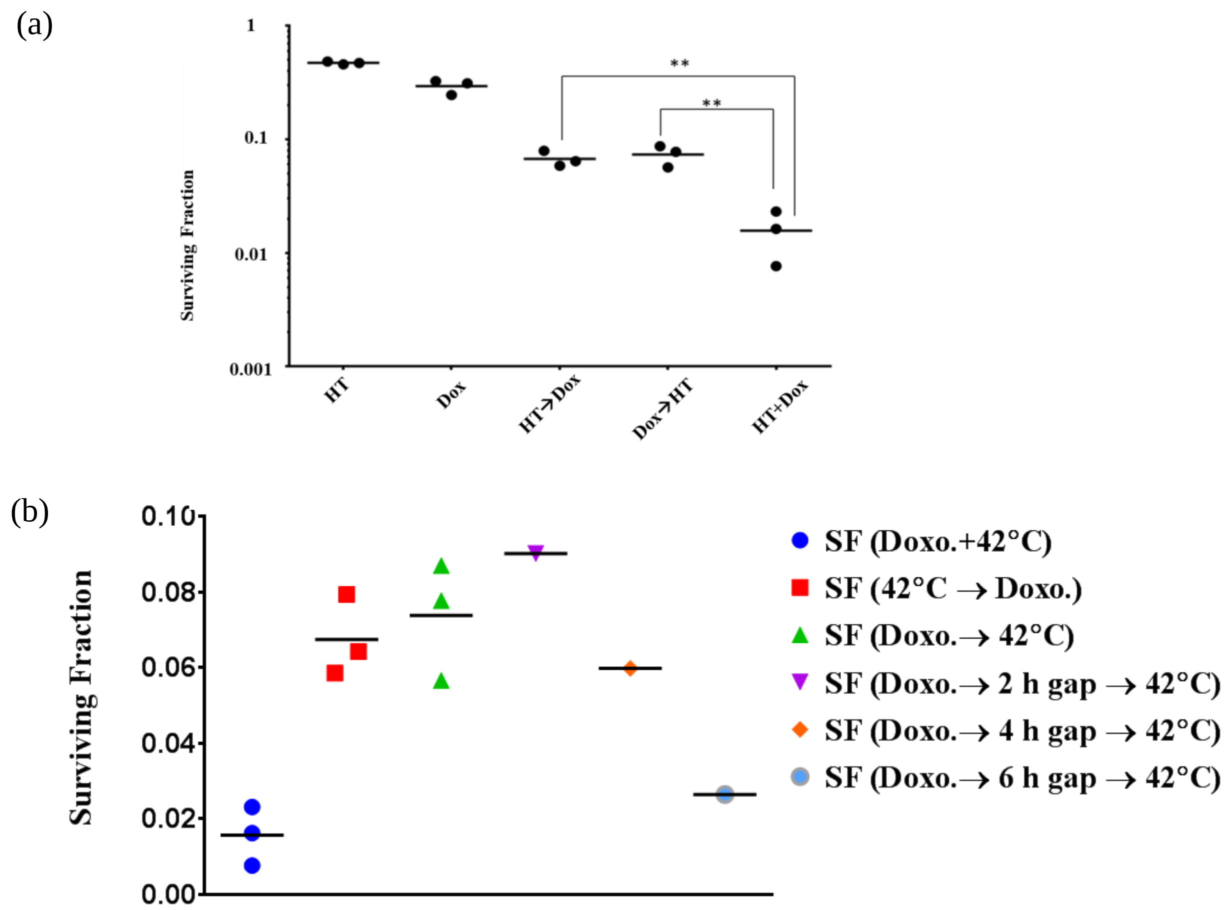

Figure S4: (a) Univariate scatterplot showing measured clonogenic survival of HCT116 cells following varied exposures to heat stress and doxorubicin combinations. For each, individual data points are plotted following exposure to 42ºC for 60 minutes (HT) and doxorubicin concentration of 0.5µg/ml for 90 minutes (Dox). From various sequences of heat-stress (42ºC) and doxorubicin (0.5µg/ml) administration, simultaneous application of both modalities yielded the greatest cytotoxicity. (* $p < 0.002$, one-way ANOVA with post-hoc Tukey's multiple comparisons test). Effect of combination schedules containing a time-gap between subsequent Dox and HT treatments on clonogenic survival of HCT116 cells is shown in (b).

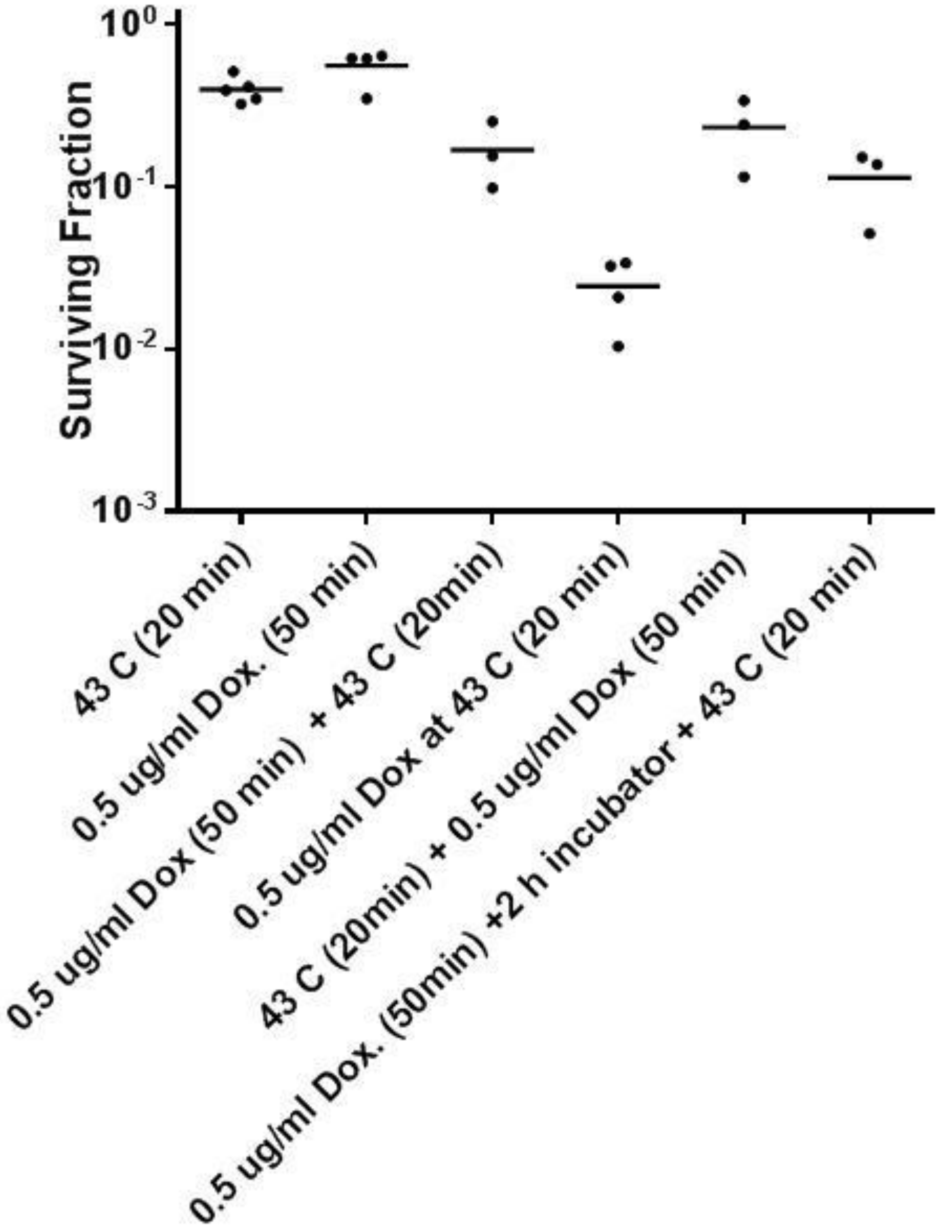

Figure S5: Univariate scatterplot showing measured clonogenic survival of HCT116 cells following varied exposures to heat stress and doxorubicin combinations. For each, individual data points are plotted following exposure to 43ºC for 20 minutes (HT) and doxorubicin concentration of 0.5µg/ml for 50 minutes (Dox).

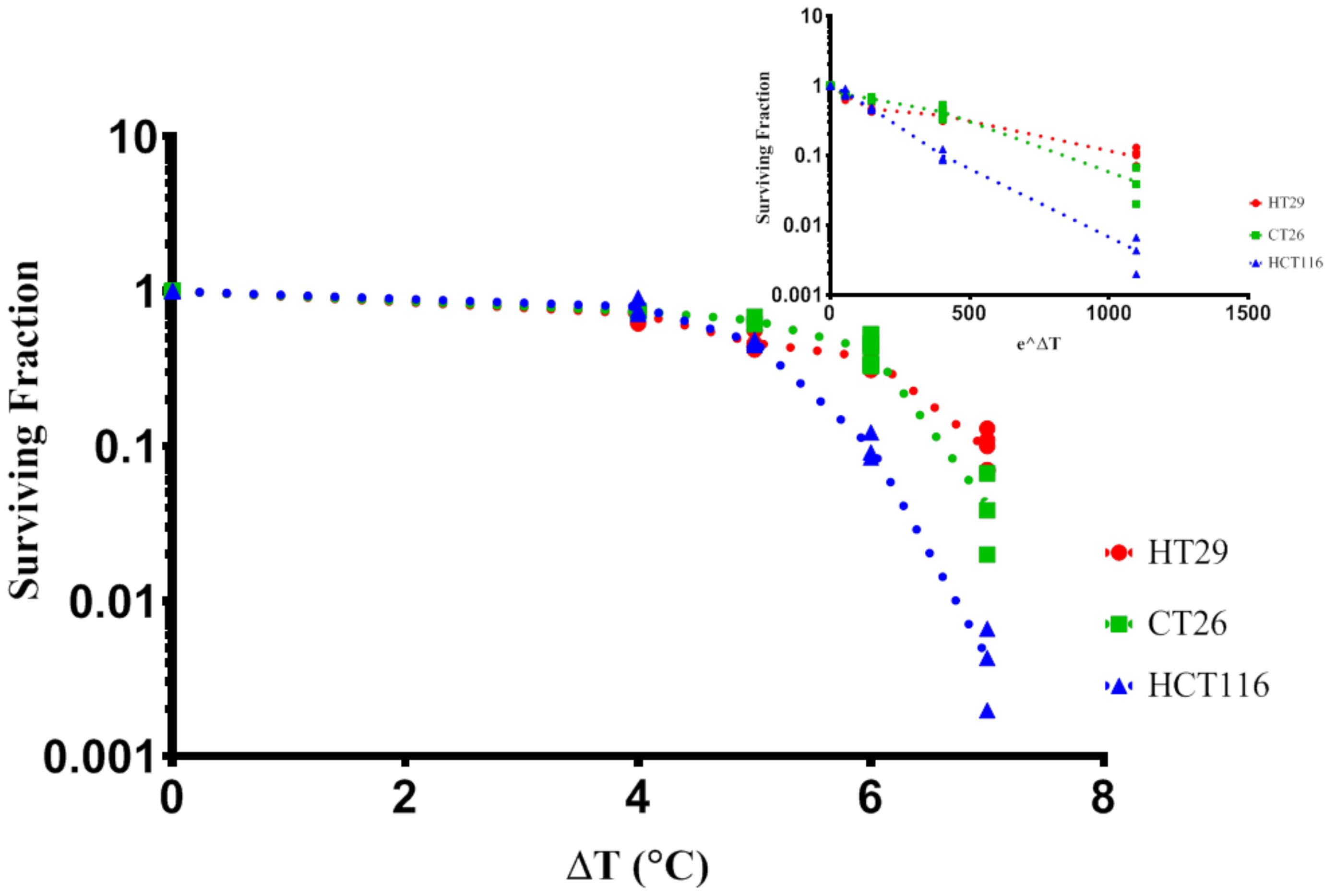

Figure S6 Clonogenic survival of HCT116 (▲), HT29 (●) and CT26 (■) cells following heat-stress (single agent HT) with escalating temperature difference, $\Delta T = T - T_0$, where T_0 is the reference temperature 37ºC. Inset shows data with abscissa plotted as $e^{(\Delta T)}$, reflecting the Arrhenius relationship of response to elevated temperature.

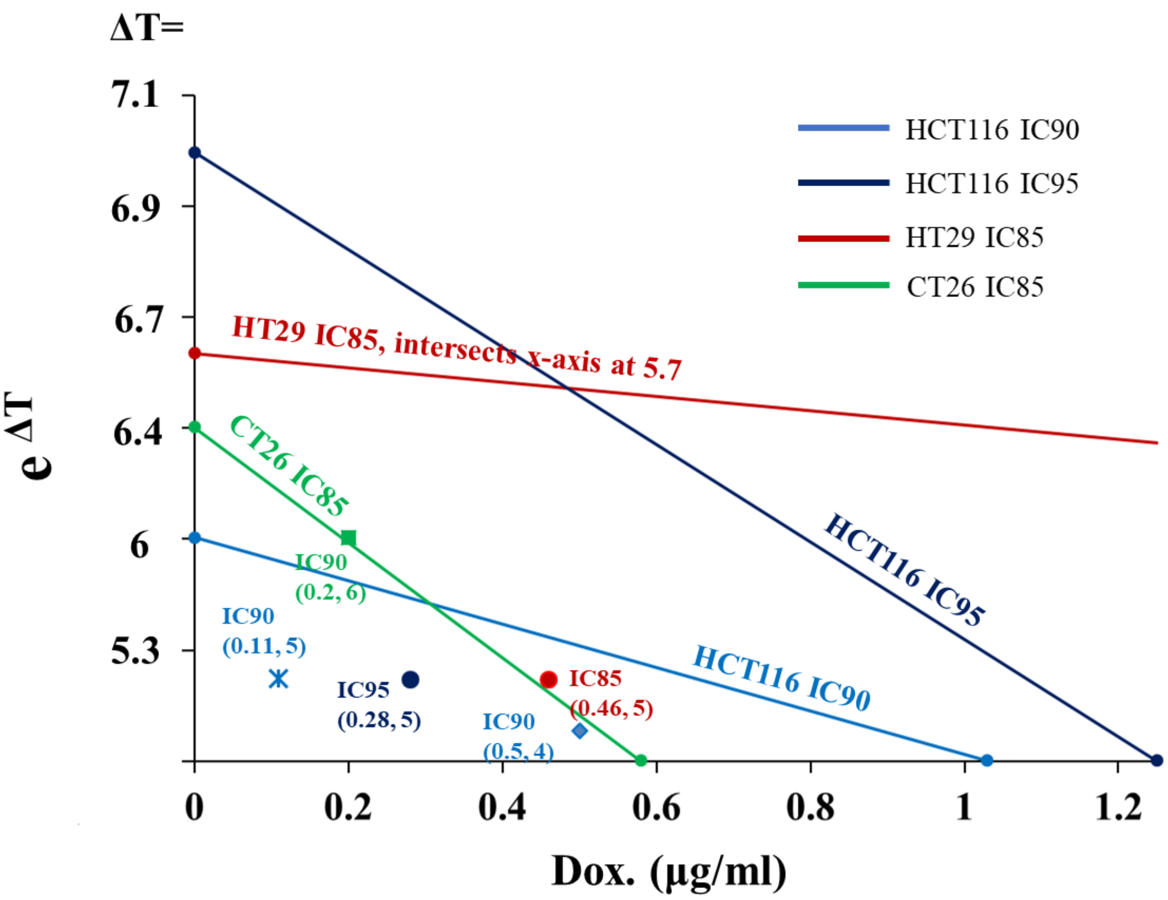

Figure S7. Isobologram analysis of doxorubicin dose escalation and $e^{(\Delta T)}$, where $\Delta T = T\text{-}T_0$, is the temperature difference in HT treatments with respect to T_0 = 37°C, to determine whether combination treatments were synergistic, additive, or antagonistic. The lower left region (origin) of the IC90 line is the synergistic region and the upper right region represents antagonistic interactions. All data points that lie on the line indicate additive effects of the combination. Combined exposure to doxorubicin and heat stress produces effects, measured by clonogenic survival, that indicate synergistic interactions. *(x,y) represent Dox dose and HT ΔT used in combination treatments.
